# A collaborative process for developing a weight management toolkit for general practitioners in Australia—an intervention development study using the Knowledge To Action framework

**DOI:** 10.1186/s40814-016-0060-4

**Published:** 2016-04-23

**Authors:** Elizabeth Ann Sturgiss, Kirsty Douglas

**Affiliations:** Academic Unit of General Practice, Australian National University Medical School, Canberra, Australia

**Keywords:** Obesity, Family practice, Health promotion, Knowledge To Action

## Abstract

**Background:**

Obesity is commonly seen in the Australian general practice population; however, few resources are specifically targeted at GPs working with these patients. The National Health and Medical Research Council (Australia) guideline for managing patients who are overweight and obese supports the involvement of a regular health professional. As 85 % of the population visit a GP annually, resources to support GPs working with this patient population are needed.

This study describes the collaborative process used to develop an obesity management programme based on current Australian guidelines for GPs and their patients to be used in primary care. The Knowledge To Action framework was applied to develop a weight management toolkit for GPs based on current Australian guidelines. This draft was then reviewed by clinical GPs, GP registrars, consumer representatives and allied health professionals using focus groups and interviews. The participants gave feedback on the content, layout and acceptability of the documents. The feedback from the stakeholder groups was evaluated, and changes were incorporated into the final documents. A graphic designer was contracted to assist with the layout to improve useability and attractiveness of the documents.

**Results:**

A total of 38 participants gave feedback on the draft weight management programme, and the research team amalgamated their responses to further improve the documents. The general response from GPs and consumer representatives was positive with most conveying their wish to try the programme themselves.

**Conclusions:**

“The Change Program” is a practical tool for Australian GPs to use with their patients who are overweight or obese. It was developed in collaboration with GPs, allied health professionals and consumer stakeholders based on current Australian guidelines. It is currently being piloted in five general practices.

## Background

An increasing proportion of patients who see GPs are overweight or obese [1, 2], and there are currently no weight management programmes that can be delivered solely by a GP in primary care in Australia [3]. Lifestyle interventions to reduce weight in primary care have had varying degrees of success in the first 12 months with most showing a return to previous weight after that time [4–7]. There are few primary care interventions that involve a family doctor [8], and most require referral to an outside practitioner or lifestyle coach. There is some evidence that patients who are satisfied that their primary care practitioner is involved in the weight loss intervention lose more weight [9]. In Australia, it is recognised that as the number of people who are obese increases, we need good tools to support GPs as the first point of contact in the healthcare system [3].

Australian guidelines suggest that GPs should be involved in identifying patients, assessing their health risk and then referring to a multidisciplinary team as needed whilst acting as a care co-ordinator [10]. It is suggested that GPs put together a management plan for their patients but there is minimal direction as to the exact content of such a plan. The guidelines focus on three areas [10]: nutrition, physical activity and behavioural interventions. We have previously published our findings from synthesising and amalgamating the recommendations from current guidelines [11].

Patients are keen for their GP to be involved in both weight management [12] and giving nutrition advice [13]. Despite this information from patients, there are few weight management interventions that involve the GP in the actual intervention. For patients that wish to work with their GP on weight management, there are few resources to guide them and there are no specific programmes. Patients may be unable to access multidisciplinary care for a variety of reasons—cost [14], availability and preference [12]. As obesity affects more people within a population, it is important to have as many options available for patient choice as possible.

This intervention development study [15] describes the method and outcome of the collaborative process we used to develop an obesity management programme based on current Australian guidelines for GPs and their patients to be used in primary care. A weight management programme gives suggestions to the GP as to how often they should see their patient, the appropriate content of consultations and direction for areas to be discussed with the patient.

**Fig. 1:**
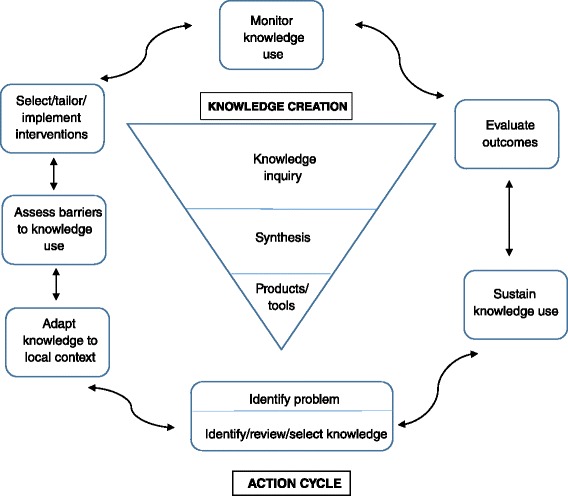
Knowledge To Action diagram. This diagram depicts the phases of the KTA framework with the “action cycles” in *rectangles* surrounding the “knowledge creation” phase in a *triangle*. Adapted from Graham, I.D., et al. [18]

Our process was informed by Fransen et al. [16] in their development of a minimal intervention strategy for primary care patients in The Netherlands. Guided by the Knowledge To Action (KTA) framework [17], we developed programme materials using principles of co-creation with stakeholders. The KTA is a knowledge exchange framework that assists in ensuring guidelines are relevant to local organisational and cultural conditions. The aim of the framework is to reduce the gap between the evidence base and clinical practice by making guidelines and resources that are produced in a collaborative fashion with end users and other interested parties. The framework has two main parts: initially, the “knowledge funnel” is used to collate current expertise into a usable form such as guidelines, and then the “action cycles” are used in an iterative process to ensure the knowledge is relevant and practical to the local context. The framework is a cyclical one, best described by the diagram from the original work by Graham et al. (see Fig. 1) [18].

Our aim is to provide GPs with evidence-based weight management resources to be used with their patients in primary care. By describing the process of developing this complex intervention, we hope to assist others who are planning similar interventions in general practice which aligns with the principles of dissemination outlined in the Medical Research Council’s guidelines for developing complex interventions [19]. We also discuss the utility of the KTA framework to develop tools to be used in the clinical decision space based on recommended guidelines.

## Methods

The knowledge enquiry and synthesis phase of the KTA framework involved four clinical GPs synthesising selected Australia current guidelines [11]. This synthesis resulted in the development of an initial draft that included a GP handbook as well as a patient workbook (see Table 1). The patient workbook was developed based on self-management principles which aim to enhance a person’s ability to care for themselves and thereby reduce the consequences of living with a chronic condition [20]. All patient worksheets were written to maximise readability. We used the Simple Measure of Gobbledygook (SMOG) [21] readability index and aimed for a SMOG of 8 (equivalent to a grade 6 standard of reading) wherever possible.

**Table 1:** Contents of the GP handbook and patient workbook

GP handbook:	
1. Welcome	
2. Who is this programme for?	
3. Work up	
4. Why is it so hard to lose weight?	
5. Nutrition	
6. Physical activity	
7. Behavioural interventions to support weight loss	
8. Trouble shooting and communication	
9. Medical causes for obesity	
Patient workbook:	
1. Welcome	
2. Upcoming appointments	
3. Goal setting	
4. Measurements	
5. Nutrition	
6. Physical activity	
7. Behavioural supports	
8. Physical activity diary	
9. Nutrition diary	
10. Relapse prevention	

The three action phases of the KTA framework (adapt knowledge to local context, assess barriers/supports to knowledge use, tailor intervention) were then undertaken using qualitative methodologies. Our participants included:

- General practitioners
- Training GP registrars
- Consumer representatives who are trained volunteers who aim to promote the consumer (patient) voice within the healthcare system
- Representative bodies for chronic illness which are advocacy and research organisations that aim to reduce the impact of the specific disease that they represent
- Allied health professionals including dieticians and psychologists

We used purposive sampling and continued to interview both GPs and consumer representatives until no new issues were raised. To recruit a diverse sample of participants, we contacted GPs through a number of avenues: via the Australian Capital Territory Medicare Local (primary health network group), through Practice-Based Research Network contacts, at local GP grand round events and convenience sampling via colleague networks. Consumer representatives were contacted via the Health Care Consumers’ Association in our local region. The Association used their regular processes for asking their members to give us feedback. We also contacted relevant representative bodies for chronic illnesses associated with obesity in our local region. These organisations were asked to comment on the documents with reference to their particular area of expertise.

Three investigators who are all clinical GPs with BMI 20–25 acted as interviewers, and an outline of the topics that were discussed is given in Table 2. The investigators asked the participants to look through the books, to give feedback freely as they went and also directed particular comment on certain sections (see Table 2). Only one investigator attended each interview/focus group for approximately 1 hour in all instances. All the participants were interviewed at their place of work or at a venue that they most preferred.

**Table 2:** Outline of feedback sought from GP and consumer representatives

Consumer feedback	
1. Logistics including frequency of suggested appointments	
2. Layout and name	
3. Graphics and presentation	
4. Goal setting page	
a. Is the language appropriate?	
b. Is it clear how to use the goal setting?	
5. Overall impression	
a. Would you like to try it?	
General practitioner feedback	
1. Logistics and information	
a. Time commitment	
b. Frequency of appointments	
c. Is there information you would like that is missing?	
d. Would you like an education programme that is aligned with this programme?	
2. Layout	
3. Graphics	
4. Indexing	
a. Any obvious things missing from the index	
5. Overall impression	
a. Would you like to try it?	

All groups reviewed both the patient and GP books, except for the consumer representatives who reviewed only the patient workbook. The participants were given the books at the start of the session, apart from the allied health representatives who had access to the material prior to the interview for a detailed review. Some feedback was audiotaped and transcribed, and others had detailed notes and writings on the actual research materials. This distinction was dependent on the wishes of the participant and the noise levels at the location of the interview.

During the interviews and focus groups, the interviewers checked understanding with the participants by summarising points raised and checking for accuracy. After each interview or focus group, the research team met to review the data that was collected. Data from transcriptions was analysed for themes. At any point where there was new feedback, or a feedback that was opposite to the previous feedback, the research team discussed how to incorporate it based on current guidelines. The graphic designer was asked to incorporate all the suggested changes from the participants. Finally, the documents were reviewed by a local psychologist with a special interest in obesity care as well as by a local dietician to ensure the information provided was accurate and complete.

This study was approved by the Australian National University Human Research Ethics Committee protocol number 2014/055, and the participants signed the consent forms prior to giving their feedback.

## Results

A total of 38 participants gave feedback on the programme materials, and the details of the participants are given in Table 3.

**Table 3:** Details of participants

Participant	Form of feedback	Total number
General practitioner	One-on-one interview	4
GP registrar	Focus group	1 group with 14 attendees
General practitioner	Focus group	3 groups (3 GPs, 4 GPs, 4 GPs)
Healthcare consumer representative	One-on-one interview	5
Representative bodies for chronic illness	One-on-one interview	2
Dietician	One-on-one interview	1
Psychologist	One-on-one interview	1
		Total—38

### Knowledge creation

The contents of each of the books are outlined in Table 1. Building on the recommendations of Fransen et al. [16], we involved a graphic designer early in the process to ensure that the layout and useability of the documents were maximised. The name of the programme, “The Change Program”, was developed by the four clinical GPs. The team wanted a name that sounded hopeful, did not overly emphasise weight and built an idea that “lifestyle change” was needed for better outcomes.

### Recruitment process

Recruitment for this research proceeded smoothly and easily. For the GPs, recruitment was most successful via email through the general practice academic unit of the medical school. This was more successful than newsletter invitations or promotion at grand round meetings. The GP registrars were approached via email on two occasions and were asked to volunteer to attend their training day early to give feedback. The recruitment of consumer representatives occurred with only one email to the Health Care Consumers’ Association who then instigated their usual processes for asking their volunteers to be involved. This ease of recruitment reflects the genuine interest in the management of obesity in primary care in our local community. A few of the participants from each of the stakeholder groups have remained part of our research and now sit on our research advisory committee.

### Action phases

A majority of participants thought the programme looked useable at face value.

…this is a great idea, and I think the GP's need to be more involved in the whole conversation about weight loss, 'cause I think in a lot of cases it's something that it's too delicate, and what do I say, and what if they get upset, and so nothing is said. (Representative body 1)

This was especially so in the GP and GP trainee groups with most asking if they could keep a copy of the programme materials after their interview.

I think having something substantial that you can give to patients is a really good idea [discussing patient handbook] (GP registrar)

### Assess barriers/supports to knowledge use

The GPs all stated that they would want an interface that interacted with their computer software.

That’s always handy, if simply on the screen, 'cause you look at them and then you do the things with the patient and then you fill in your notes afterwards. And that's really great. (GP registrar)

Based on this feedback, we developed a template that could be adapted for the different programmes used in our region. This provided a place to record appointment information and gave the GP prompts for factsheets to refer the patient to in their workbook.

None of the GPs interviewed wanted an education programme associated with the toolkit.

Would you like an education program that is aligned with this program’s delivery?

Not sure we would go. Isn’t that the point of the workbook? (self-explanatory) (GP)

They described feeling overwhelmed at times with the number of education events they were invited to participate in. They wanted a set of resources that could be referred to as needed, and they felt that there was enough information in the handbook for them to be able to assist a patient.

None of the consumer representatives thought that the programme looked like an unworkable idea. There were some concerns about the logistics of the programme including cost (both monetary and time) to the patient and the feasibility of implementing it within general practice.

- wonders about practicalities i.e. would it be practical to get into their GP that often? (consumer rep 1)
- thinks will depend upon flexibility of the GP and wonders how likely is it that the GP will invest the time or whether it would be sustainable for the GP and wonders if there would be implications if program not followed (consumer rep 2)
  Field notes from the interviewer

The stakeholder representatives were not as positive about the programme. There was concern that GPs would not be able to implement the programme, that GPs would lose focus on other important health condition management and that perhaps patients would not want to see their GP for this sort of advice.

“Is it realistic to even think that people would use their GP as someone who would help them in their weight loss? Or would they be also looking at a dietician to do the same thing? Or a coach?” (Representative body 1)

“And I'd be really concerned if that happened to my patients, that they be on a six to 12 month treatment programme to sort their obesity out and then no-one looked at their [chronic illness] in the meantime, and they were allowed to continue to have high blood sugars” (Representative body 2)

This feedback was quite opposite from what we saw from the GP and consumer participants.

I love this book [patient handbook]. And if I had just this book it would change the way I practice I think, just to have a go to for… I like it a lot. (GP registrar)

Both the GPs and consumer participants were concerned about the cost to the patient.

The GP's don't have a lot of time for things like this. So I'm just sort of wondering here what would that look like for the patient? Is this something that they would be paying for themselves? Or is this something that would be covered? Because I think it always seemed to come down to how expensive, how much is it going to cost for me to be able to do this? (Representative body 1)

### Adapt knowledge to local context

One group of GPs who worked with a defined vulnerable population felt that the programme would not be helpful in their population and would need modification for their population group. If their population wanted to be involved in a programme like this, our team would offer to meet and tailor the programme as needed. From their experience working in general primary care, they thought it would be useful in that setting.

Some GPs felt that there was not enough prescriptive information in the programme and they would like more exact direction on how to structure each programme. Other GPs liked the “looser” nature of the set-up and felt that this allowed them to work with what they knew about their patient and their community. We took this on board and developed a consultation schedule that had suggested topics and actions for each visit. We had this in the front of the GP handbook should any practitioner feel they wanted this level of direction.

The booklet's not forcing you to do all this at the same time or anything, it's just saying at… over a period of time. So you've got freedom as the GP to decide as you like. So if you judge the patient is really throwing this at them up front is just going to put a roadblock in the way straight away (GP registrar)

### Tailor intervention

Most GPs wanted more nutrition information particularly relating calories eaten to the amount of physical activity needed to burn it off. This was added as a new factsheet in the patient workbook.

Some consumer representatives were worried that there was too much text and the layout was not appealing. This feedback was acted on; readability was re-evaluated, text boxes were added and more graphics were inserted.

- thinks “really good, crisp and clear”; thinks too dense (too much writing) and needs more breakout boxes and pictures (consumer rep 1)
- Too much information to take in – needs more pictures; Not much variation in colour or graphics; “Looks boring and overwhelming (consumer rep 2)
  Field notes from the interviewer

I like the idea though that everything is in the booklet format, this is their little bible that they can use. (Representative body 1)

The graphic designer was involved in making approximately ten different versions of the documents following the participants’ feedback to incorporate the changes suggested. The final feedback on the draft was sought from a dietician and psychologist who both have a special interest and expertise in obesity. Both found that the information in the programme was correct for their discipline-specific background. The psychologist was particularly impressed at the detail around behavioural interventions as they usually find this is lacking in many current weight management programmes.

The dietician also felt that there was not enough nutrition information and was a little surprised at the focus on psychological interventions. They felt that we had a lot of information telling the patient what not to eat but not enough about good foods to eat. From this, we included examples of daily menus that were consistent with dietary guidelines.

The next step for “The Change Program” is a pilot implementation trial based on Normalisation Process Theory [22] to assess feasibility, useability and acceptability to both GPs and patients.

## Discussion

By using a collaborative process such as this, we aim to produce a toolkit for weight management in primary care that is acceptable to both patients and GPs. Obesity is currently not being recognised and managed in primary care as much as guidelines would recommend [3]. If we increase the treatment choices available to patients and empower GPs with structured tools to be used, we can improve the likelihood that obesity will be managed within the primary care setting. As discussed previously, as GPs are the first point of contact with the health system, they have good reach into the community and need supportive tools for management [3].

Our data has shown a keen interest from GPs and consumer representatives on the role of GPs in managing obesity in primary care. Representatives from chronic illness organisations were less positive about the overall feasibility of such a weight management programme in general practice. They were reflecting from a perspective outside of the relationship between a GP and a patient using their experience in management. We have taken the views of the GPs and consumers as more reflective of the population likely to use the programme. Although it is possible that they were influenced to give positive answers as they were interviewed by GPs, it is unlikely that every person interviewed was similarly influenced and we received some negative feedback from GPs on aspects of the programme that could be improved. This is an example of the importance of reaching for feedback from multiple sources especially those at the frontline to ensure their perspectives are not missed.

Involving a graphic designer from the beginning of the intervention development meant that our materials looked attractive and easy to use. We were able to use the skills of the graphic designer to incorporate changes when we had feedback about the layout of the materials. We would recommend working with a designer that is happy to work via email, is accessible and is responsive to changes suggested by your team.

The process for developing intervention studies is not described very often in the literature [15]. By outlining the details of the collaborative process we utilised, interested parties are able to trace the origins of the weight management toolkit and what stakeholders had input. It is also important for processes to be published so that other researchers can learn from our experience in developing this complex intervention. Through transparent reporting of development processes, it is possible that research waste can be reduced by stopping repetition of similar interventions or mistakes [15].

By starting with national guidelines for the management of patients who are overweight and obese in primary care [10], we have attempted to make our toolkit generalisable to the Australian context. The stakeholders involved in the action phases of our research were all drawn from our local region. The Australian Capital Territory (ACT) has a population that has a higher than average income compared to the rest of Australia. The ACT also has the lowest rate of “bulk-billing” for general practitioner services where the entire consultation cost is covered by the national health insurance [23]. It is possible that the feedback from our local region is not generalisable to a national level.

Using the KTA framework to describe the development of clinical practice guidelines is well established [24]. Our process informs a further use of the KTA framework where the knowledge creation process begins with identification of guidelines that are then synthesised. The initial knowledge creation process is completed with the development of tools that can be used in the clinical decision-making process. The action phases are used to strengthen and develop the tools prior to the implementation of the intervention. These initial action phases with feedback from relevant stakeholders allow for some problems with interventions to be identified prior to the pilot-testing phase and for further testing of interest in participants for the research project.

This co-creation with all relevant bodies and individuals encourages ownership and interest in the research project. Poor recruitment and response rates within research, especially of GPs, are often described with resultant research waste [25]. Strategies to improve recruitment and retention of GPs usually discuss methods of contact, incentivising and having a colleague send the invitation [26, 27]. However, co-creation with practitioners is not mentioned as a method for enhancing ownership, acceptance and support of research. Our method of co-creation with GPs involved in meaningful ways in early intervention development is likely to enhance recruitment and participation.

## Conclusion

By involving multiple different stakeholder groups, we were able to produce programme materials for weight management in primary care to be used by GPs in consultation with their patients using a Knowledge To Action framework. This process led to multiple changes in our weight management materials including changes to layout for readability, more detailed information on nutrition and more explicit instructions for the frequency and content of appointments.

This programme supports increasing calls for increased general practice involvement in obesity management as the first point of call in the health system and having the greatest reach into the community. The interest of the primary care community and patients is testament to the ongoing research that is needed to better support GPs in their management role for this difficult health condition. These programme materials are now being used in an implementation pilot study in five general practices in the next step to assessing clinical effectiveness of such a programme.

## Abbreviations

BMI: body mass index
GP: general practitioner
KTA: Knowledge To Action
